# Utero-placental calcium and magnesium ion channels: A systematic review of obstetric implications of their alterations

**DOI:** 10.1080/19336950.2026.2724674

**Published:** 2026-08-26

**Authors:** Gaudence Ndinganire, Aime Patrick Niyomugabo, Daniel Udofia Owu, Makinde Vincent Olubiyi, David Chibuike Ikwuka, Alphonse Niyodusenga, Mayowa Jeremiah Adeniyi, Muluken Walle, Onyinye Cynthia Okeke, Abdul-Rahuf Aderemi Feyitimi, Ejike Daniel Eze, Akaninyene Ubong Ime, Elemi John Ani, Abdulazeez Jimoh, Abdullahi Hussein Umar, Diresebachew Wondimu Haile, Abdullateef Isiaka Alagbonsi, Olufunke Onaadepo

**Affiliations:** Department of Medical Physiology, School of Medicine and Pharmacy, College of Medicine and Health Sciences, University of Rwanda, Huye, Rwanda

**Keywords:** Calcium channels, magnesium regulation, myometrium, placenta, pregnancy complications

## Abstract

Despite the established roles of calcium (Ca^2+^) and magnesium (Mg^2+^) in placental function and uterine contractility, limited information exists on how dysregulation of major ion channels contributes to poor pregnancy outcomes. We synthesized data on the consequences of Ca^2+^ and Mg^2+^ channelopathies in uterine and placental functions. Using PubMed, Wiley Online, AJOL, and Web of Science databases for article search, a systematic review of forty-nine papers published between 2000 and March 2026 was carried out and reported in accordance with the PRISMA 2020 guideline. Based on the PICO framework, eligible studies involving human, animal, and in vitro designs were chosen and subjected to narrative analysis. L-type and T-type voltage-gated Ca^2+^ channels, together with transient receptor potential channels, emerged as principal mediators of placental Ca^2+^ transport and myometrial contractility. Mechanosensitive Piezo1 channels mediate stretch-activated Ca^2+^ influx, while store-operated Ca^2+^ entry pathways involving STIM1–Orai1 sustain intracellular Ca^2+^ homeostasis. Potassium–Ca^2+^ coupling channels modulated membrane hyperpolarization and anti-labor effects, and intracellular regulators such as PMCA and RYR1 fine-tuned Ca^2+^ homeostasis. The Mg^2+^ transporters are essential for preserving Mg^2+^ homeostasis and regulating Ca^2+^-dependent excitability. Dysregulation of these ion channel systems was consistently linked to abnormal uterine contractility, preterm birth, preeclampsia, fetal growth restriction, and adverse pregnancy outcomes. Both Ca^2+^ and Mg^2+^ ion channelopathies represent both a potential therapeutic target and a mechanistic factor underlying key obstetric complications.

## GRAPHICAL ABSTRACT

**Figure:**
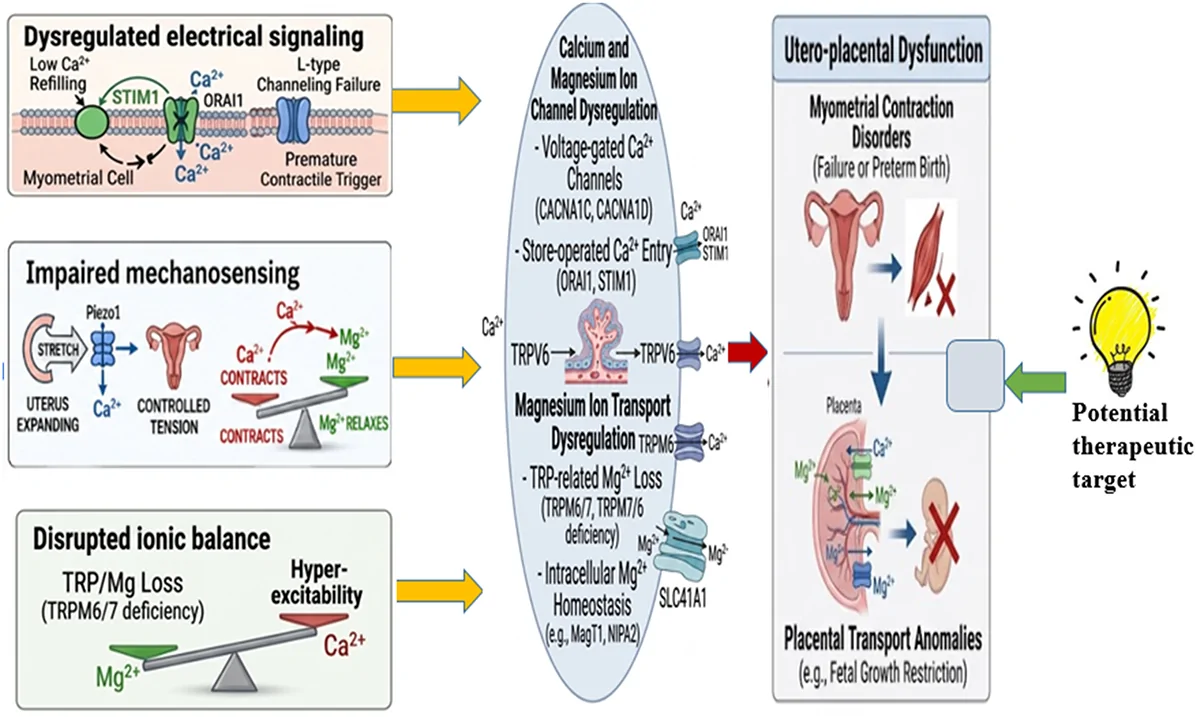

## Introduction

Smooth muscle contraction is a fundamental physiological mechanism that controls the operations of several organ systems, including the gastrointestinal, circulatory, respiratory, and reproductive systems [1,2]. Among these, the uterus and placenta are particularly crucial during pregnancy, where adequate uteroplacental blood flow, uterine quiescence, and healthy delivery depend on vascular regulation and properly synchronized smooth muscle activity. Research in uterine and placental physiology has traditionally focused on hormonal and paracrine regulations, particularly the roles of oxytocin, prostaglandins, progesterone withdrawal, and estrogen stimulation [3,4]. While these pathways are important, cellular electrophysiology shows that ion channel activity is the final common pathway driving myometrial contraction, just like in male gonadal function [5,6], even though most previous studies focused on the endocrine pathway [7–18]. For instance, estrogen has been demonstrated to increase T-type calcium (Ca^2+^) channel activity, thereby enhancing spontaneous uterine contractions [4,19]. Prostaglandins (particularly F2α [PGF2α] and E2 [PGE2]) also elicit Ca^2+^-mediated uterine contractions by promoting intracellular Ca^2+^ mobilization [20,21] through the production of inositol triphosphate (IP_3_) that links metabolic and electrical inputs to mechanical contraction [22]. Then, Ca^2+^ binds to calmodulin to activate myosin light-chain kinase, initiating actin–myosin cross-bridge cycling and generating contractile force that regulates uterine tone and parturition [1,2]. Overall, placental function and uterine contractility are fundamental physiological processes required for successful pregnancy, fetal development, and parturition, and they both depend on precisely regulated intracellular Ca^2+^ signaling [20].

Maternal–fetal magnesium (Mg^2+^) transfer occurs primarily through the placenta, ensuring fetal requirements for skeletal formation, neuromuscular transmission, protein synthesis, cellular metabolism, and nervous system maturation are met [23,24]. Mg^2+^ acts as a physiological antagonist to Ca^2+^ by blocking voltage-gated Ca^2+^ channels, regulating transient receptor potential (TRP) channels and other ion transport pathways, preventing excessive Ca^2+^ influx and shielding tissues from hyperexcitability [25–27]. In the uterus, the myometrium remains in a state of low excitability and minimal contractile activity during pregnancy to facilitate fetal growth but becomes highly excitable and contractile as labor draws near, necessitating careful control of ion channels that drive Ca^2+^ signaling and membrane depolarization [19,28,29]. The Ca^2+^-Mg^2+^ signaling balance is crucial for controlling fetal perfusion, placental vascular resistance, uterine contractility, and optimal fetal development throughout gestation [26,30–32], and disturbances in Mg^2+^ homeostasis have been associated with uterine hypercontractility or hypocontractility, preterm labor, hypertensive disorders of pregnancy, placental insufficiency syndromes, fetal growth restriction, and altered placental blood flow [33]. In addition, reduced intracellular Mg^2+^ may enhance Ca^2+^-mediated smooth muscle contraction and contribute to increased uterine sensitivity, whereas Mg^2+^ deficiency has also been linked to inflammatory activation and oxidative stress during pregnancy [34].

The main pathways for Ca^2+^ entry during myometrial depolarization are voltage-gated Ca^2+^ channels (VGCCs), such as L-type and T-type channels [4,27,35]. Recent evidence also highlights mechanosensitive channels, such as Piezo1, as contributors to myometrial contraction [36–39]. Furthermore, sustained Ca^2+^ influx, placental Ca^2+^ transport, and myometrial contraction are all made possible by store-operated Ca^2+^ entry (SOCE) channels like Orai1 and TRP family members like TRPV6 and TRPC3 [40–42]. Mechanical stretch and shear stress activate Piezo1, producing Ca^2+^ influx that promotes myometrial contractility, inflammatory signaling, endothelial responses, trophoblast fusion, and placental development, with potential implications for the initiation of labor [38,43–45]. In fetoplacental endothelial cells, Piezo1 acts as a mechanosensor whose altered function is associated with preeclamptic pregnancies [43,44]. Likewise, Ca^2+^-activated potassium channels, including small-conductance (SK3) and large-conductance (BKCa) channels, regulate membrane potential by promoting potassium (K^+^) efflux during normal and inflammatory conditions, thereby reducing voltage-gated Ca^2+^ entry and limiting uterine excitability [46–52]. Estradiol was shown to suppress SK3 channel expression, suggesting hormonal regulation of uterine excitability [50], while BKCa expression also differed by uterine region and gestational stage, showing region-specific and pregnancy-dependent regulation patterns [49]. ATP-sensitive potassium channel (KATP) is also expressed during pregnancy, and its activation causes membrane hyperpolarization, reducing VGCC-mediated Ca^2+^ entry and promoting uterine relaxation [53].

In contrast to uterine ion channels that primarily regulate cellular excitability (VGCCs, TRPC, BKCa, SK3, Piezo1), placental Ca^2+^ transport systems are specialized for maternal–fetal mineral transfer. Among these, TRPV6 is the principal epithelial Ca^2+^ channel responsible for transplacental Ca^2+^ uptake required for fetal skeletal mineralization [32,37,42,54,55]. The Mg^2+^ transporters, including TRPM6, TRPM7, and SLC41A1, similarly regulate placental Mg^2+^ homeostasis, trophoblast function, and fetal development [56–58]. These transporters differ functionally from channels involved in uterine contraction because their primary role is mineral transport rather than regulation of membrane excitability. Thus, maternal–fetal Ca^2+^ transfer must be controlled for proper fetal skeletal development and endocrine balance, and this homeostasis is jeopardized by dysregulation of Ca^2+^ channels in placental tissues, whether due to genetic variations (such as TRPV6 deficiency) or pathological conditions (such as hypoxia, preeclampsia) [28,32,42,59–61]. Dysregulation of placental Ca^2+^ channels may indirectly contribute to abnormal uterine contractility by disrupting placental Ca^2+^ transport, trophoblast signaling, and fetoplacental homeostasis, which influence the biochemical environment associated with the onset and progression of labor. However, direct mechanistic evidence linking placental Ca^2+^ channelopathy to uterine hypercontractility or hypocontractility remains limited [56,62,63]. Moreover, ion channelopathies have been linked to placental insufficiency syndromes, early labor, and fibroid-related overexpression of Ca^2+^ channels [64,65]. However, most uterine and placental studies reveal secondary ion channel dysregulation, which may be brought on by hormonal modulation, inflammatory signaling, hypoxia, or drug effects, rather than true congenital channelopathies. So, primary genetic defects and secondary dysregulation must be distinguished to understand mechanisms and possible treatment applications [66,67].

Currently, there is a therapeutic gap as the available obstetric treatments mostly target hormonal or receptor-mediated mechanisms rather than upstream ion channel dysfunctions. The development of new, channel-targeted interventions, as well as a deeper comprehension of labor physiology, preterm birth, and placental insufficiency, may benefit from the establishment of a mechanistic framework connecting molecular Ca^2+^–Mg^2+^ channelopathies to clinical uterine and placental disorders [36]. Despite increasing evidence, the roles of specific Ca^2+^ and Mg^2+^ channels in female reproductive tissues remain insufficiently characterized. Existing studies largely emphasized hormonal regulation of uterine activity or focused on isolated ion channel families, leaving the integrated functions of Ca^2+^-Mg^2+^ channel systems across uterine and placental tissues inadequately explored [31,68]. Furthermore, the interactions between mechanical stretch signaling, electrophysiological pathways, and Mg^2+^-dependent modulation of Ca^2+^ influx are not clearly defined. Therefore, this systematic review aims to synthesize current evidence on these interconnected mechanisms and clarify how Ca^2+^–Mg^2+^ channel systems influence uterine contractility, placental mineral transport, and pregnancy outcomes.

## Methodology

### Study design

To compile disparate molecular, physiological, and clinical information, this study conducted a systematic review of the literature and reported it according to the Preferred Reporting Items for Systematic Reviews and Meta-Analyses (PRISMA) 2020 guidelines [69], as adopted in previous systematic reviews [70–81]. The PRISMA 2020 checklist can be found in the Supplementary File S1.

### Search strategy

A comprehensive and systematic literature search of PubMed, Web of Science, AJOL, and Wiley Online Library was conducted for studies published between 2000 and March 2026 on Ca^2+^ and Mg^2+^ channel expression, regulation, transport, and dysfunction in uterine and placental tissues during pregnancy, including both physiological functions and pathological alterations linked to obstetric disorders such as preterm labor, preeclampsia, uterine atony, and fetal growth restriction. Recognizing the need for comprehensive literature capture, the search strategy was substantially broadened to include studies investigating Ca^2+^ OR Mg^2+^ channels independently, as well as those examining both. The search incorporated extensive free-text terms and MeSH headings for all the major ion channel families, including VGCCs (L-type, T-type), TRP channels (TRPC, TRPV, TRPM, TRPV4, TRPV6, TRPM6, TRPM7), SOCE/CRAC channels (STIM1, Orai1), Mg^2+^ transporters (SLC41A1, MagT1), mechanosensitive channels (Piezo1), potassium channels (BKCa, SK3, KATP), intracellular Ca^2+^ channels (IP3Rs, RyRs), and Ca^2+^-sensing receptors (CaSR). A sample of the terms combination adapted for most of the databases is: ((“calcium” OR “Ca^2+^” OR “magnesium” OR “Mg^2+^” OR “calcium channels” OR “L-type calcium channel” OR “T-type calcium channel” OR “TRP channels” OR “TRPC” OR “TRPV” OR “TRPM” OR “TRPV6” OR “TRPM6” OR “TRPM7” OR “MagT1” OR “store-operated calcium entry” OR “SOCE” OR “CRAC” OR “Orai1” OR “STIM1” OR “calcium-sensing receptor” OR “CaSR” OR “Piezo1” OR “BKCa” OR “SK3” OR “SLC41A1” OR “IP3 receptor” OR “ryanodine receptor”) AND (“uterus” OR “myometrium” OR “placenta” OR “trophoblast” OR “syncytiotrophoblast”) AND (“pregnancy” OR “preterm labor” OR “preeclampsia” OR “uterine atony” OR “fetal growth restriction” OR “labor” OR “parturition”)).

The search strategy was adapted to the indexing system and syntax of each database while maintaining the same conceptual framework. Reference lists of all included studies and relevant review articles were also manually screened to identify any additional eligible publications not retrieved through the electronic database searches. However, the authors acknowledge that no search strategy can guarantee complete capture of all relevant literature, and some relevant studies might have been inadvertently missed despite the broadened search terms and manual reference screening. The full search strings for each database are presented as Supplementary File S2.

### Inclusion and exclusion criteria

After studies were imported into a reference management system (Mendeley), duplicates were removed, and they were evaluated using the PICO-based eligibility criteria (Table 1) in two stages (title/abstract and full text). Articles were deemed “irrelevant” if they did not address Ca^2+^ or Mg^2+^ channel function in uterine or placental tissues, lacked mechanistic or functional data, or focused on non-reproductive tissues without clear relevance to uteroplacental physiology. Studies that only mentioned Ca^2+^ or Mg^2+^ in passing without specific channel-related investigations were also excluded.

**Table 1.** Inclusion and exclusion criteria based on PICO framework.

PICO component / category	Inclusion criteria	Exclusion criteria
Population (P)	Human, mammalian animal, and in vitro studies investigating uterine, myometrial, placental, trophoblast, syncytiotrophoblast, or fetoplacental endothelial tissues.	Studies involving non-reproductive tissues or organ systems without relevance to uterine or placental physiology; studies on plants, microorganisms, or non-mammalian species.
Intervention / Exposure (I)	Studies investigating Ca^2+^ and/or Mg^2+^ channels or transporters, including VGCCs, TRP channels, Piezo1, SOCE (STIM1/Orai1), PMCA, RYR1, BKCa, SK3, KATP, TRPV6, TRPM6/TRPM7, SLC41A1, and related pathways; including altered expression, function, mutations, or pharmacological modulation.	Studies addressing general Ca^2+^ or Mg^2+^ metabolism without investigating ion channels or transport mechanisms; studies unrelated to uteroplacental ion channel function.
Comparison (C)	Healthy versus pathological pregnancy, pregnant versus non-pregnant tissues, normal versus altered channel expression/function, treated versus untreated groups, wild-type versus knockout/mutant models, or appropriate experimental controls.	Studies lacking an appropriate comparison or control group to evaluate ion channel function or regulation.
Outcomes (O)	Outcomes related to uterine contractility, excitation–contraction coupling, intracellular Ca^2+^/Mg^2+^ regulation, mechanotransduction, placental Ca^2+^ or Mg^2+^ transport, trophoblast function, placental vascular regulation, fetal mineralization, or pregnancy complications (e.g. preterm labor, preeclampsia, uterine atony, fetal growth restriction).	Outcomes unrelated to uteroplacental physiology or lacking mechanistic or functional relevance to Ca^2+^ or Mg^2+^ ion channels.
Study Design	Original peer-reviewed experimental, observational, genetic, pharmacological, animal, and in vitro studies reporting primary data.	Review articles, systematic reviews, meta-analyses, editorials, conference abstracts, letters, protocols, and studies without primary experimental data.
Time Frame	Articles published between January 2000 and March 2026	Publications outside the specified period.
Language	Peer-reviewed articles published in English.	Non-English publications and non-peer-reviewed literature.

### Data extraction

Important molecular, physiological, and clinical data on Ca^2+^ and Mg^2+^ channel functions in uterine and placental tissues were extracted, along with study parameters, model type, ion channel categories, and experimental procedures. The results were organized thematically by ion channel type and tissue context to enable an integrated analysis of their functions in uterine contractility, placental transport, and obstetric issues, such as preterm labor, preeclampsia, uterine atony, and fetal growth limitation, and were compiled and presented in Supplementary File S3. Due to the significant heterogeneity observed across the included studies in terms of methodologies, species, and reported outcomes, a narrative synthesis approach was adopted to systematically analyze and integrate the findings [82–86].

### Risk of bias assessment

The Systematic Review Center for Laboratory Animal Experimentation (SYRCLE), Office of Health Assessment and Translation (OHAT), and Science in Risk Assessment and Policy evaluation framework (SciRAP) Risk of Bias (RoB) tools were used because the review encompassed various study types, necessitating a design-specific approach to ensure accurate assessment. Because the appraisal criteria differed according to study design, each study was evaluated using the checklist appropriate for its methodology rather than applying identical criteria across all studies. The SYRCLE tool was applied to animal studies, the OHAT was used for human observational studies, while the SciRAP was used for human in vitro/ex vivo studies, allowing for a comprehensive and appropriate evaluation of study quality and risk of bias [87]. To guarantee objectivity and dependability, each study was evaluated separately by two reviewers. Disagreements between reviewers were resolved through discussion until consensus was achieved. Where consensus could not be reached, a third reviewer adjudicated the final decision. Randomization, blinding, allocation concealment, outcome data completeness, selective reporting, and possible additional sources of bias were among the evaluation’s main focal areas. Studies were classified as Low Risk (✓), High Risk (✗), or Unclear Risk (?) for each domain (Supplementary File S4).

## Results and discussion

### Study characteristics

**Figure 1.**
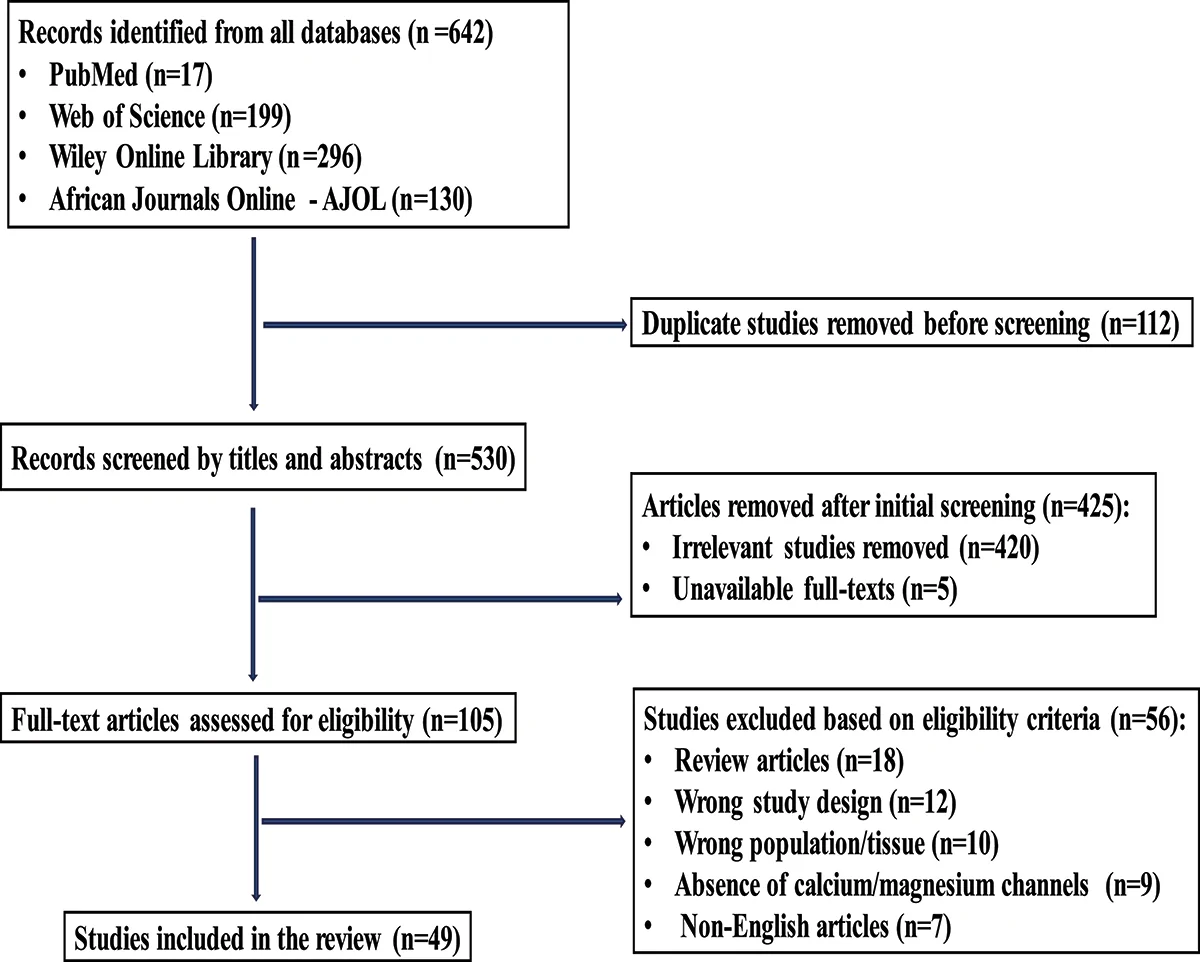
PRISMA flow chart of the article selection process.

A total of 642 records were identified through a systematic search of electronic databases, out of which 112 duplicate records were removed before screening, leading to the screening of 530 studies based on titles and abstracts. Of these, 425 articles were excluded during title and abstract screening, including 420 that were not relevant to the study objectives and 5 whose full texts were not available. After full-text evaluation of the remaining 105 articles, 56 were excluded for not meeting the eligibility criteria, such as being review articles (*n* = 18), wrong study design (*n* = 12), population/tissue (*n* = 10), absence of Ca^2+^ and Mg^2+^ channels (*n* = 9), and non-English articles (*n* = 7). The systematic review was ultimately based on 49 studies that satisfied the inclusion criteria (Figure 1). Overall, most included studies were classified as having an intermediate (moderate) risk of bias, reflecting the presence of methodological limitations in some appraisal domains. These include unclear reporting of randomization and blinding, and concerns regarding sample selection or comparator groups in some observational studies. However, intervention/exposure measurement was consistently rated as low risk in most studies, supporting the reliability of the primary experimental findings.

The 49 studies included in this systematic review comprised a heterogeneous mix of research designs, including experimental in vitro studies (*n* = 32), in vivo animal studies (*n* = 13), and observational human studies (*n* = 4). Most of the experimental in vitro studies investigated mechanistic pathways under controlled laboratory conditions, whereas animal studies predominantly used mouse and rat models to explore uterine contractility, placental development, and maternal–fetal ion transport. Human studies mainly evaluated ion-channel expression and pregnancy-associated molecular alterations. Geographically, the studies originated predominantly from Europe (*n* = 24), followed by North America (*n* = 14) and Asia (*n* = 11) (Table 2).

**Table 2.** Mechanistic classification and characteristics of included studies investigating uteroplacental Ca^2+^ and Mg^2+^ ion channels (*n* = 49 studies).

Mechanistic category	Study	Experimental model	Key findings
TRP Channels (*n* = 15)	[88]	Human myometrium	TRPC and TRPV channels contribute to the regulation of uterine contractility via Ca^2+^ influx and excitation–contraction coupling
	[89]	Pig myometrium	TRPV4 regulates uterine smooth muscle function via mechanosensitive Ca^2+^ signaling
	[90]	Human myometrial cells	TRPV4 mediates diacylglycerol-induced Ca^2+^ signaling
	[91]	Pregnant human uterus	TRP channels contribute to smooth muscle activation in term pregnant human myometrium
	[40]	Human myometrial biopsies	TRPC expression is influenced by cytokines and is important during labor
	[92]	Human myometrial biopsies	Uterine stretch modulates uterine growth and contractility in pregnancy via alterations in TRPC-mediated Ca^2+^ signaling
	[111]	Mouse placenta	TRP channels are involved in the placentation process
	[94]	Mouse uterus	TRPV6 contributes to uterine Ca^2+^ regulation
	[95]	Bovine uterus and placenta	TRPV6 expression varied according to pregnancy stage; altered TRPV6 regulation may affect Ca^2+^ availability
	[32]	Knockout mice	TRPV6 is involved in maternal-fetal Ca^2+^ transport
	[96]	Human placenta	TRPV6 mutation interferes with maternal-fetal Ca^2+^ transport through the placenta
	[97]	Rat myometrium	TRPV4 regulates uterine tone and contractile activity; mechano-sensation contributes to uterine function via mechanosensitive Ca^2+^ signaling; TRPV4 dysfunction may disturb uterine tone
	[64]	Human uterine fibroid tissues	High expression of TRPs contributes to fibroids in patients
	[61]	Pregnant rat placenta	TRP expression in the placenta is regulated by hypoxia during pregnancy
	[114]	Pregnant human myometrium	TRPC1 is involved in nifedipine-induced contraction in myometrium
VGCCs (*n* = 6)	[98]	Human uterine myometrium	Labor and non-labor myometrium express different subtypes of Ca^2+^ transporter genes
	[99]	Telocytes from human myometrium	VGCC expression in the telocytes of the myometrium is regulated by estradiol during pregnancy
	[35]	Human myometrial cells	VGCC polymorphism contributes to hypercontractility and preterm birth
	[19]	Pregnant rat uterine smooth muscle	T-type Ca^2+^ channels are expressed in the pregnant rat myometrium and play a role in the regulation of phasic contraction
	[101]	Human placental tissue from preeclamptic high-altitude pregnancies	Reduced placental *CACNA1C* expression suggests impaired Ca^2+^ channel regulation associated with pre-eclampsia and altered placental adaptation
	[100]	Murine uterine myometrium	Identified Ca^2+^ channel regulators involved in uterine contraction; explains excitation–contraction coupling in labor; channel abnormalities may affect uterine contractile function
K^+^–Ca^2+^ Coupling (*n* = 7)	[47]	Rat uterus	BKCa is expressed in the mouse myometrial smooth muscle, and its inhibition suppresses spontaneous and LPD-stimulated uterine contraction
	[48]	Uterine strips from genetically altered mice	SK3 regulates uterine function by limiting influx through L-type Ca^2+^ channels
	[49]	Myometrial biopsies from pregnant and non-pregnant women	Expression of BKCa subunits in pregnant myometrium is reduced during labor
	[46]	Mice	SK3 channel activity must be downregulated for gravid uterus to generate labor contractions sufficient for delivery in both term and preterm mice
	[50]	Rat uterus	SK3 channel is present in the uterine endometrium and myometrium and is regulated by estradiol
	[52]	Myometrium samples from term pregnant women	BKCa channel and its immunomodulatory partners regulate Ca^2+^ dynamics in human myometrial smooth muscle cells
	[53]	Human myometrium biopsies	Functional KATP channel subunits are expressed in pregnant myometrium, and their downregulation may contribute to enhanced uterine contractility associated with onset of labor
Placental Ca^2+^ Transport (*n* = 6)	[103]	DNA samples from a neonate with rare bone disease and her parents	TRPV6 variants interfere with fetoplacental Ca^2+^ transfer crucial for in utero skeletal development
	[59]	Human placental cells	Ca^2+^ homeostasis and transport through placental are compromised in preeclamptic pregnancies
	[104]	Mice placenta	Placental Ca^2+^ transport adaptations are different in normal and growth-restricted fetuses
	[105]	Human trophoblasts	Ca^2+^ transporters are involved in the basal Ca^2+^ uptake by the syncytiotrophoblasts
	[42]	Human placental cells	TRPV6 controls the extracellular matrix structure of the placental labyrinth
	[54]	Human placenta	Cyclophilin B is an accessory protein of TRPV6 channel protein and is involved in its activation and Ca^2+^ uptake.
Magnesium Transport (*n* = 5)	[57]	Adult mouse model	TRPM6 regulates Mg^2+^ homeostasis and embryonic development, prenatal development, and survival at adulthood
	[58]	Human placental cells	SLC41A1 is overexpressed in preeclamptic placentas
	[26]	Human myometrial samples	The tocolytic effect of magnesium sulfate is dependent on an extracellular mechanism
	[102]	Mouse model	Mg^2+^ deficiency during pregnancy impairs placental development and fetal growth
	[56]	Human placental cells	Expression of Mg^2+^/inorganic phosphate channels is downregulated in pre-eclamptic placental tissues during preterm labor and under hypoxia
SOCE (*n* = 4)	[106]	Human myometrial cells	SOCE channels are expressed in the human myometrium and participate in human myometrial Ca^2+^ signaling during pregnancy
	[107]	Human myometrial cells	SOCE may be an important route for Ca^2+^ entry into the syncytiotrophoblast of term, but not first-trimester placentas
	[108]	Human myometrial cells	STIM1 and ORAI1-ORAI3 contribute to intracellular Ca^2+^ dynamics in human myometrial cells
	[41]	Human myometrium	There are changes in the Orai1 protein expression and activity of human myometrial CRAC channels in term-pregnant, laboring myometrium
Piezo1 (*n* = 4)	[43]	Human placental endothelial cells	Piezo1 regulates the mechano-transduction of fetoplacental endothelial cells
	[38]	Human uterine tissues	Piezo1 acts as a critical regulator of uterine function, and its overexpression may predispose to preterm labor through heightened myometrial activity and inflammation
	[44]	Human placental cells	Piezo1 regulates placental vascular adaptation via endothelial mechano-transduction
	[45]	Mouse model	Piezo1 contributes to Ca^2+^ influx and uterine contraction during labor
Intracellular Ca^2+^ Regulation (*n* = 2)	[109]	Human myometrial cells	RYR1 regulates intracellular Ca^2+^ release from the sarcoplasmic reticulum, affecting excitation–contraction coupling, and its mutations alter Ca^2+^ regulation and muscle contraction control
	[110]	Outbred white non-pregnant rats	Changes in the Ca^2+^ signal and contractile activity in uterine myocytes involve PMCA

Abbreviations: Ca^2+^, calcium ion; Mg^2+^, magnesium ion; TRP, transient receptor potential; TRPC, canonical TRP channel; TRPV, vanilloid TRP channel; TRPM, melastatin TRP channel; VGCC, voltage-gated calcium channel; BKCa, large-conductance calcium-activated potassium channel; SK3, small-conductance calcium-activated potassium channel 3; KATP, ATP-sensitive potassium channel; SOCE, store-operated calcium entry; STIM1, stromal interaction molecule 1; Orai1, calcium release-activated calcium channel protein 1; Piezo1, mechanosensitive ion channel; PMCA, plasma membrane calcium ATPase; RYR1, ryanodine receptor 1.

The Ca^2+^ and Mg^2+^ channels involved in uteroplacental physiology were classified into multiple mechanistic groups. The TRP channels (TRPC, TRPV, and TRPM; *n* = 13) represented the largest category and were implicated in uterine excitability, trophoblast proliferation, maternal–fetal Ca^2+^ transport, placental mineralization, mechano-transduction, and Mg^2+^ homeostasis [32,40,88–97]. The VGCCs (*n* = 6), including L-type and T-type channels, were associated with excitation–contraction coupling, labor-associated Ca^2+^ influx, preterm labor, fibroid-related hypercontractility, placental Ca^2+^ signaling, and pre-eclampsia [19,35,98–101]. The K^+^-Ca^2+^ coupling pathways (BKCa, SK3, and KATP; *n* = 7) regulate membrane potential, intracellular Ca^2+^ oscillations, uterine quiescence, and labor-associated contractility [46–50,52,53]. The Mg^2+^ transport pathways (*n* = 5), involving TRPM6, TRPM7, and SLC41A1, maintain intracellular Mg^2+^ homeostasis, regulate uterine excitability, and support placental function and fetal development [26,56–58,102]. Placental Ca^2+^ transport mechanisms (*n* = 6), predominantly mediated by TRPV6, regulate maternal–fetal Ca^2+^ transfer, trophoblast differentiation, and fetal skeletal mineralization [42,54,59,103–105]. The SOCE (STIM1, Orai1, and TRPC1; *n* = 4) regulates sustained intracellular Ca^2+^ influx and myometrial contraction [41,106–108]. Piezo1 mechanosensitive channels (*n* = 4) mediate stretch-induced Ca^2+^ influx involved in mechano-transduction, trophoblast fusion, placental vascular adaptation, and uterine contraction during labor [38,43–45]. Finally, intracellular Ca^2+^-regulatory mechanisms (*n* = 2) involving PMCA and RYR1 maintain intracellular Ca^2+^ homeostasis through Ca^2+^ extrusion and sarcoplasmic reticulum Ca^2+^ release [109,110]. Collectively, these findings demonstrate that Ca^2+^ and Mg^2+^ channels regulate uterine excitability, excitation–contraction coupling, placental Ca^2+^ and Mg^2+^ transport, trophoblast function, vascular adaptation and maternal–fetal Ca^2+^ homeostasis, and that their dysregulation contributes to preterm labor, pre-eclampsia, fetal growth restriction, abnormal uterine contractility, and impaired fetal skeletal mineralization.

### Ion channels and transporters in myometrial and placental physiology

**Figure 2.**
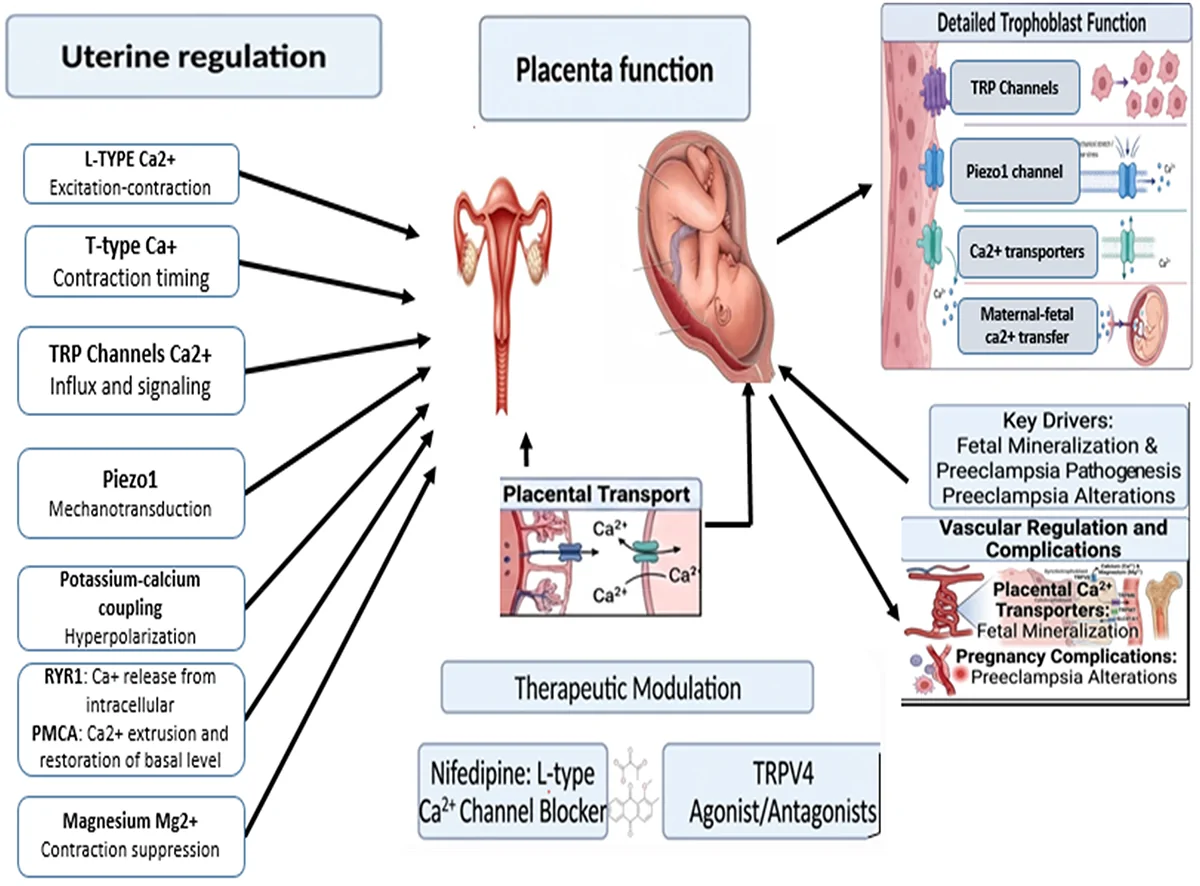
Mechanistic map of calcium (Ca^2+^)–magnesium (Mg^2+^) channelopathies in uterine and placental functions. This schematic illustrates the integrated network of ion channels and transporters regulating myometrial excitability, uterine contractility, and placental mineral transport. In the uterus, L-type and T-type voltage-gated Ca^2+^ channels regulate excitation–contraction coupling and contraction timing, while TRP channels and Piezo1 mediate receptor-operated and mechanosensitive Ca^2+^ signaling. Potassium (K^+^)–Ca^2+^ coupling pathways involving SK3, BKCa, and KATP channels promote membrane hyperpolarization and regulate uterine relaxation. The SOCE pathways (STIM1/Orai1) sustain prolonged calcium entry, whereas Mg^2+^ transport pathways counterbalance Ca^2+^-dependent excitability. In the placenta, TRP channels contribute to trophoblast function and Ca^2+^ signaling, while TRPV6 and related transporters regulate maternal–fetal Ca^2+^ transfer required for fetal mineralization. Dysregulation of these pathways contributes to preterm labor, preeclampsia, fetal growth restriction, and impaired uteroplacental vascular function.

The reviewed evidence demonstrates that uteroplacental physiology is regulated by an integrated network of Ca^2+^ and Mg^2+^ channels rather than by individual channel families acting independently. Interactions among TRP channels, VGCCs, K^+^–Ca^2+^ coupling pathways, Mg^2+^ transporters, SOCE components, Piezo1, and intracellular Ca^2+^ regulators coordinate uterine excitability, trophoblast function, placental mineral transport, vascular adaptation, and maternal–fetal Ca^2+^ homeostasis. Dysregulation of these pathways contributes to major obstetric complications, including preterm labor, pre-eclampsia, fetal growth restriction, abnormal uterine contractility, and impaired fetal skeletal mineralization, highlighting their potential as therapeutic targets and biomarkers for adverse pregnancy outcomes (Figure 2).

#### TRP channels

The TRP channels constituted the largest mechanistic category (*n* = 15 studies) and collectively demonstrated broad roles in uterine and placental physiology. The TRPC channel mediates receptor- and cytokine-induced Ca^2+^ entry that regulates myometrial excitability and inflammatory signaling during labor [40,88,91,92]. TRPV4 functions as a mechanosensitive channel controlling stretch-induced Ca^2+^ influx and uterine tone [89,90,97], whereas TRPV2, TRPM6, and TRPM7 were consistently identified as the principal placental Ca^2+^ transporters supporting placentation, maternal–fetal Ca^2+^ transfer, and fetal skeletal mineralization [94,95,111]. TRPM7 additionally regulates intracellular Mg^2+^ homeostasis, trophoblast proliferation, and placental development [32,96]. Collectively, these findings indicate that TRP channels integrate Ca^2+^ and Mg^2+^ signaling across multiple reproductive tissues rather than functioning as isolated transport proteins. Their involvement in mechano-transduction, trophoblast biology, and placental mineral transport suggests that dysregulation of TRP channels may simultaneously impair uterine contractility and placental function, thereby contributing to preterm labor, fetal growth restriction, and defective placental development.

#### VGCCs

Six studies investigated L-type and T-type VGCCs, demonstrating their central role in excitation–contraction coupling. The L-type channels mediate the Ca^2+^ influx required for labor-associated uterine contractions [98]. Additional studies have reported hormonal regulation of VGCC expression during pregnancy [19,99,100], altered Ca^2+^ signaling in pregnancy complications [62], and reduced placental *CACNA1C* (Ca_v_1.2) expression in pre-eclamptic pregnancies at high altitude [101]. These observations reinforce VGCCs as the principal pathway for Ca^2+^ entry initiating uterine contraction, and their altered expression or function may therefore contribute not only to abnormal myometrial contractility but also to impaired placental Ca^2+^ signaling. The identification of placental *CACNA1C* dysregulation further suggests that abnormal Ca^2+^ channel expression may represent a molecular mechanism linking placental dysfunction with hypertensive disorders of pregnancy.

#### K^+^–Ca^2+^ pathways

Seven studies evaluated BKCa, SK3, and KATP channels as regulators of membrane potential and Ca^2+^-dependent contraction. The BKCa channels control intracellular Ca^2+^ oscillations, promote uterine relaxation, and regulate labor-associated contractility [47,49,52]. The SK3 channel contributes to membrane hyperpolarization and reduced myometrial excitability and is regulated by estradiol during pregnancy [46,48,50], while KATP activation promotes uterine relaxation [53]. Whereas VGCCs promote Ca^2+^ entry and contraction, K^+^ channels reduce membrane depolarization and limit Ca^2+^ influx, thereby preventing premature uterine activation and highlighting the complementary relationship between K^+^ and Ca^2+^ channels. Disruption of this balance may contribute to uterine hypercontractility and spontaneous preterm labor.

#### Placental Ca^2+^ transport mechanisms

Six studies focused specifically on placental Ca^2+^ transport, predominantly mediated by TRPV6. These investigations consistently demonstrated that TRPV6 regulates maternal–fetal Ca^2+^ transfer, trophoblast differentiation, placental adaptation, and fetal skeletal mineralization [42,54,59,103–105]. The evidence indicates that efficient placental Ca^2+^ transport is essential for normal fetal development. Impaired TRPV6-mediated Ca^2+^ transfer may compromise fetal mineralization while simultaneously altering placental function, suggesting that placental Ca^2+^ transport represents an important pathway linking maternal Ca^2+^ homeostasis with fetal outcomes.

#### Mg^2+^ transport pathways

Five studies investigated Mg^2+^ transport mechanisms involving TRPM6, TRPM7, and SLC41A1. TRPM7 regulates placental development and embryonic survival [57,102], whereas SLC41A1 maintains intracellular Mg^2+^ homeostasis [26,58]. Mg^2+^-dependent modulation of Ca^2+^ signaling reduces uterine contractility [26] while TRPM6 and TRPM7 dysregulation are associated with pre-eclampsia [56] and impaired placental development [102]. These studies support the concept that Mg^2+^ homeostasis directly influences Ca^2+^-dependent uterine function. Rather than acting independently, Mg^2+^ appears to regulate Ca^2+^ signaling through multiple transport pathways. This mechanistic interaction provides biological support for the clinical use of magnesium sulfate (MgSO_4_) in preventing excessive uterine excitability and managing pregnancy complications such as pre-eclampsia.

#### Store-operated Ca^2+^ entry (SOCE)

Four studies examined STIM1-, Orai1- and TRPC1-mediated SOCE pathways. These studies demonstrated that depletion of intracellular Ca^2+^ stores activates sustained Ca^2+^ influx required to maintain prolonged myometrial contractions and intracellular Ca^2+^ homeostasis [41,106–108]. Although most available evidence derives from in vitro models, the findings consistently demonstrate that SOCE complements VGCC-mediated Ca^2+^ entry by maintaining prolonged contractile activity. This suggests that pharmacological modulation of SOCE components may represent an additional therapeutic strategy for disorders characterized by abnormal uterine contractility.

#### Piezo1 mechanosensitive channels

Four studies investigated Piezo1 as a mechanosensitive Ca^2+^ channel. Piezo1-mediated stretch-induced Ca^2+^ influx was shown to be involved in placental endothelium mechano-transduction [43], trophoblast fusion [38], placental endothelial function and vascular adaptation [44], and uterine contraction during labor [45]. Compared with other channel families, evidence for Piezo1 remains relatively recent but is rapidly expanding. The consistent observation that mechanical stretch activates intracellular Ca^2+^ signaling suggests that Piezo1 may function as an important sensor linking uterine distension, placental vascular adaptation, and initiation of labor.

#### Intracellular Ca^2+^-regulatory mechanisms

Two studies examined intracellular Ca^2+^ regulation through PMCA and RYR1. RYR1 regulated Ca^2+^ release from the sarcoplasmic reticulum during contraction [109], while PMCA maintained intracellular Ca^2+^ homeostasis by extruding Ca^2+^ from myometrial cells [110]. Although comparatively few studies addressed intracellular Ca^2+^ regulation directly, these findings demonstrate that intracellular Ca^2+^ handling is essential for maintaining coordinated excitation–contraction coupling. Future studies integrating PMCA and RYR signaling with plasma membrane Ca^2+^ channels may provide a more complete understanding of uterine Ca^2+^ dynamics during pregnancy and labor.

### Altered ion channel expression in normal pregnancy and pregnancy complications

#### Physiological pregnancy adaptation

Normal pregnancy requires tightly coordinated regulation of Ca^2+^ and Mg^2+^ channels to maintain uterine quiescence while supporting placental development and fetal growth. Evidence from the included studies demonstrated dynamic regulation of multiple ion channel families throughout gestation. TRP expression increases during pregnancy and facilitates placentation, maternal–fetal Ca^2+^ transfer necessary for placental function and fetal skeletal mineralization [54,94,103,111]. TRPV4-mediated mechanosensitive signaling contributes to the regulation of uterine tone and adaptation to increasing mechanical stretch as pregnancy progresses [89,90]. Simultaneously, BKCa, SK3, and KATP channels stabilize the myometrial membrane potential, limiting excessive Ca^2+^ influx and maintaining uterine relaxation [47,48,50]. The SOCE pathways involving STIM1, Orai1, and TRPC1 further maintain intracellular Ca^2+^ homeostasis during prolonged physiological activity [106–108]. Together, these findings demonstrate that normal pregnancy depends on coordinated interactions among Ca^2+^ and Mg^2+^ transport systems to preserve uterine quiescence while preparing the uterus and placenta for labor.

#### Preterm labor

Several studies demonstrated that dysregulation of Ca^2+^-permeable channels contributes to premature activation of the myometrium. Increased expression and activity of L-type VGCCs enhanced Ca^2+^ influx and promoted excitation–contraction coupling during labor and fibroid [64,98]. Inflammatory stimulation also alters TRPC expression and increases Ca^2+^ signaling within the myometrium, suggesting that inflammatory pathways may trigger premature uterine activation [40]. Mechanosensitive Piezo1 channels also promote stretch-induced Ca^2+^ influx and inflammatory signaling that enhance uterine contractility [38]. Conversely, reduced activity of K^+^ channels, including BKCa and KATP, may diminish membrane hyperpolarization, thereby facilitating excessive Ca^2+^ entry through VGCCs [47,49]. Collectively, these findings suggest that preterm labor arises from the disruption of the normal balance between excitatory Ca^2+^ influx and inhibitory K^+^-mediated membrane stabilization, resulting in premature uterine hypercontractility.

#### Pre-eclampsia

Pre-eclampsia was consistently associated with altered expression of Ca^2+^ and Mg^2+^ transport pathways within the placenta. Placental expression of the L-type Ca^2+^ channel gene, *CACNA1C* (Ca_v_1.2), was significantly reduced in pre-eclamptic pregnancies, suggesting impaired Ca^2+^ signaling and placental adaptation [101]. Altered expression of TRPM6, TRPM7, and SLC41A1 further indicates disruption of Mg^2+^ homeostasis in placental tissues [56,58]. In addition, aberrant activation of Piezo1 and TRPV4 contributes to placental endothelial dysfunction, abnormal mechano-transduction, and vascular maladaptation [43]. Also, reduced trophoblast Ca^2+^ homeostasis was observed in pre-eclamptic placentas [59]. These findings suggest that impaired regulation of Ca^2+^ and Mg^2+^ channels contribute to placental insufficiency, endothelial dysfunction, and the increased vascular resistance characteristic of pre-eclampsia.

#### Fetal growth restriction

The TRPV6-mediated Ca^2+^ transport was essential for fetal skeletal mineralization and placental Ca^2+^ transfer [54,103]. Experimental studies have further demonstrated the adaptive regulation of placental Ca^2+^ transport pathways during fetal growth restriction, aiming to preserve the fetal Ca^2+^ supply [104]. Under chronic hypoxic conditions, altered expression of placental Ca^2+^ channels, including *CACNA1C*, suggests impaired placental adaptation and reduced Ca^2+^ signaling [101]. Together, these studies indicate that disruption of placental Ca^2+^ transport represents a common mechanism contributing to impaired fetal growth and abnormal placental function.

#### Abnormal uterine contractility and uterine fibroids

Abnormal uterine contractility results from dysregulation of multiple ion channel families rather than alterations in a single pathway. Increased expression and activity of L-type and T-type VGCCs promote excessive Ca^2+^ influx and myometrial hypercontractility during pregnancy [35,98,99]. Altered VGCC expression is associated with preterm birth (PTB), suggesting a mechanistic basis for increased contractility in this condition [35]. BKCa, SK3, and KATP channels normally oppose Ca^2+^-dependent contraction by maintaining membrane hyperpolarization and regulating intracellular Ca^2+^ oscillations [46–48,53]. Therefore, disruption of the balance between Ca^2+^ influx and K^+^-mediated membrane stabilization may contribute to uterine hypercontractility, dysfunctional labor, and fibroid-associated abnormalities. Overall, the evidence from the 49 included studies demonstrates that altered expression or function of Ca^2+^ and Mg^2+^ channels underlie the transition from physiological pregnancy to pathological conditions. While coordinated regulation of TRP channels, VGCCs, K^+^ channels, Mg^2+^ transporters, SOCE pathways, Piezo1, and intracellular Ca^2+^ regulators maintains uterine quiescence and placental homeostasis during normal gestation, dysregulation of these pathways contributes to preterm labor, pre-eclampsia, fetal growth restriction, and abnormal uterine contractility. These findings highlight ion-channel signaling as a central mechanistic pathway in obstetric disease and identify several promising targets for future therapeutic interventions and biomarker development.

### Comparisons with previous reviews and novel insights

A thorough knowledge of the interactions between these ion channel systems in uterine and placental tissues is still lacking, especially when it comes to the interplay between Ca^2+^ influx pathways, K^+^-mediated membrane stabilization, and Mg^2+^-dependent modulation [26,58]. Most previous studies have examined ion channels as separate entities, with limited attention to their functional integration, subtype diversity, and mechanosensitive regulation. In contrast, this review synthesizes evidence from 49 studies to provide an integrated perspective on uterine and placental ion channel physiology. It brings together key channel systems, including voltage-gated Ca^2+^ channels, TRP channels, Piezo1 mechanosensitive channels, K^+^ channels, and Mg^2+^ transporters, and outlines how their coordinated activity regulates myometrial excitability, uteroplacental vascular tone, and maternal–fetal ion transport [4,37,40,46,58]. Furthermore, this synthesis directly links ion channel dysregulation to major obstetric disorders such as preterm labor, preeclampsia, and impaired uterine tone, while also highlighting inconsistencies between animal models and human placental findings [103,112,113]. This provides a clearer translational perspective than previous reviews that were largely descriptive in scope. Finally, the review identifies key research gaps, particularly the underexplored role of mechanosensitive and Mg^2+^-dependent pathways, and emphasizes the need for advanced approaches such as single-cell transcriptomics and high-resolution electrophysiological mapping to better define ion channel behavior in uterine and placental tissues.

### Translational implications of the findings

The results of this review have many translational implications for research, clinical practice, and reproductive physiology. Firstly, prospective targets for pharmacologic regulation in circumstances like preterm labor or uterine hypercontractility are suggested by the consistent importance of L-type, TRP, and Piezo1 mechanosensitive Ca^2+^ channels in controlling uterine excitability and contractility [38]. In contrast to existing treatments, specific L-type channel blockers, TRP modulators, or Piezo1-targeted interventions, for instance, may serve as tocolytics with better efficacy and safety profiles, even though some off-target consequences across body tissues with Ca^2+^ channels may be imminent [97,114]. Secondly, dysregulated placental Ca^2+^ transporters, such as SLC41A1 and TRPV6, hold potential promise in prognostic and diagnostic procedures. Pregnancies at risk for preterm birth, preeclampsia, or decreased fetal Ca^2+^ supply may be identified through genetic screening for channel polymorphisms [35,103], allowing for earlier interventions or individualized monitoring plans. Furthermore, knowing how Mg^2+^ modulates uterine Ca^2+^ channels offers a physiological foundation for improving MgSO_4_ treatment and investigating new Mg^2+^-based therapies [26,113]. Furthermore, the functional mapping of TRP channels in the human endometrium offers a molecular framework for the development of novel non-hormonal contraceptives and therapeutic agents for medical pregnancy termination by interfering with Ca^2+^-dependent signaling pathways essential for embryo implantation and the maintenance of uterine quiescence [93].

### Strengths and limitations

A major strength of this review is the comprehensive evaluation of Ca^2+^ and Mg^2+^ channels across uterine and placental tissues by integrating evidence from human, animal, and in vitro studies. The review also employed PRISMA 2020 reporting guidelines together with study-specific risk-of-bias assessment tools (SYRCLE, OHAT, and SciRAP), thereby enhancing methodological rigor. However, several limitations should be acknowledged. Most included studies were experimental or animal-based, limiting direct translation to human pregnancy. Considerable heterogeneity in study designs, experimental models, and reported outcomes precluded quantitative meta-analysis. Furthermore, evidence for emerging pathways such as Piezo1, SOCE, and intracellular Ca^2+^ regulators remains limited because relatively few human studies have been conducted. Finally, restricting inclusion to English-language publications may have introduced publication and language bias.

## Conclusions

Uterine excitability, myometrial contractility, placental Ca^2+^ transfer, and overall reproductive outcomes are all regulated by Ca^2+^–Mg^2+^ channels, which include L-type, T-type, TRP, BKCa, SK3, K^+^, CRAC/Orai1, Piezo1, and placental Ca^2+^ transporters. Genetic, acquired, or pharmacologically induced dysregulation of these channels can cause aberrant parturition timing, including premature labor and fetal development restriction, as well as altered uterine contraction patterns, poor relaxation, and interrupted fetal Ca^2+^ supply. While T-type, BKCa, SK3 K^+^, CRAC/Orai1, Piezo1, and Mg^2+^-mediated pathways continue to be supported by moderate or preliminary data, primarily because of reliance on in vitro and animal research, the strongest evidence supports L-type Ca^2+^ channels and TRP channels in both myometrial and placental function. Excitation–contraction coupling, myometrial tone modulation, and maternal-fetal Ca^2+^ homeostasis are all impacted by channelopathies, according to mechanistic findings. This has significant translational implications for diagnostics, therapeutic interventions, and individualized obstetric care.

### Recommendations

Further studies are needed to verify the function of T-type, SK3 K^+^, CRAC/Orai1, Piezo1, BKCa, and Mg^2+^-mediated pathways in uterine and placental physiology through carefully planned human investigations. To treat placental malfunction and prevent preterm labor, therapies that target L-type and TRP channels also need to be investigated. There is also a need to examine genetic screening methods for Ca^2+^–Mg^2+^ channel polymorphisms to identify women who are susceptible to problems with fetal growth, abnormal labor, or poor uteroplacental Ca^2+^ transfer. The need to create integrative mechanistic models that integrate placental transport, uterine contractility, and Ca^2+^–Mg^2+^ signaling to forecast reproductive outcomes and direct tailored treatments is also worthy of future exploration. To strengthen translational potential, there is a need to address species-specific differences observed in preclinical models, particularly TRPV6 studies. Finally, subsequent studies should promote standardization of experimental designs, outcome measures, and reporting to improve evidence synthesis and enable meta-analyses.

## Supplementary Material

Revised Supplementary File S2.docx

Revised Supplementary File S4.xlsx

Revised Supplementary File S3.xlsx

Revised Supplementary File S1.docx

## Data Availability

The data used to synthesize information in this review article are available as Supplementary Files S1 to S4 submitted together with this article.
