# Supplementary material for: Utero-placental calcium and magnesium ion channels: A systematic review of obstetric implications of their alterations
Source: Channels (Austin). 2026 Aug 26;20(1):2724674. doi: 10.1080/19336950.2026.2724674 (PMC13523982; doi:10.1080/19336950.2026.2724674)
Supplement: Supplementary file 1 [file KCHL_A_2724674_SM2550.docx]

| **Section and Topic** | **Item #** | **Checklist item** | Location where item is reported |
| --- | --- | --- | --- |
| **TITLE** | | |  |
| Title | 1 | Identify the report as a systematic review. | **Utero-placental Calcium and Magnesium Ion Channels: A Systematic Review of Obstetric Implications of their Alterations** |
| **ABSTRACT** | | |  |
| Abstract | 2 | See the PRISMA 2020 for Abstracts checklist. | **Background:** Despite the established roles of calcium (Ca²⁺) and magnesium (Mg²⁺) in placental function and uterine contractility, there is limited information on how dysregulation of major ion channels contributes to poor pregnancy outcomes. We synthesized data on the consequences of Ca²⁺ and Mg²⁺ channelopathies in uterine and placental functions.  **Methods:** Using PubMed, Wiley online, AJOL, and Web of Science databases for article search, a systematic review of forty-nine papers published between 2000 and March 2026 was carried out and reported in accordance with the PRISMA 2020 guideline. Based on the PICO framework, eligible studies involving human, animal, and *in vitro* designs were chosen and subjected to narrative analysis. To ensure methodological rigor across diverse study designs, the SYRCLE tool was applied to animal studies, OHAT was used for human observational studies, and SciRAP was used for human in vitro/ex vivo studies.  **Results:**  Results from the included research consistently show that multiple ion channel systems critically regulate myometrial excitability, excitation–contraction coupling, and placental mineral transport. L-type and T-type voltage-gated Ca²⁺ channels (VGCCs), together with transient receptor potential (TRP) channels (TRPC, TRPV, TRPM), emerged as principal mediators of placental Ca²⁺ transport and myometrial contractility. Mechanosensitive Piezo1 channels mediate stretch-activated Ca²⁺ influx, while store-operated Ca²⁺ entry (SOCE) pathways involving STIM1–Orai1 sustain intracellular Ca²⁺ homeostasis. Potassium–Ca²⁺ coupling channels (SK3, BKCa, KATP) modulated membrane hyperpolarization and anti-labor effects, and intracellular regulators such as PMCA and RYR1 fine-tuned Ca²⁺ homeostasis. The Mg²⁺ transporters, including TRPM6/TRPM7 and SLC41A1, are essential for preserving Mg²⁺ homeostasis and regulating Ca²⁺-dependent excitability. Dysregulation of these ion channel systems, particularly TRP channels, VGCCs, Mg²⁺ transporters, and Piezo1, was consistently linked to abnormal uterine contractility, preterm birth, preeclampsia, fetal growth restriction, and adverse pregnancy outcomes.  **Conclusion:** Both Ca²⁺ and Mg²⁺ ion channelopathies represent both a potential therapeutic target and a mechanistic factor underlying key obstetric complications |
| **INTRODUCTION** | | |  |
| Rationale | 3 | Describe the rationale for the review in the context of existing knowledge. | Calcium (Ca²⁺) and magnesium (Mg²⁺) ions are fundamental regulators of smooth muscle contractility, vascular tone, intracellular signaling, and transplacental mineral transport during pregnancy. In uterine tissues, Ca²⁺ influx through voltage-gated and receptor-operated channels governs myometrial excitability and contraction, while Mg²⁺ acts as a physiological antagonist that stabilizes membrane potential and promotes relaxation. In the placenta, tightly coordinated calcium transport systems facilitate maternal–fetal mineral exchange essential for fetal skeletal development and growth. Dysregulation of these ion channels has been implicated in major obstetric complications, including preterm labor, preeclampsia, uterine atony, and fetal growth restriction. Although individual studies have examined specific calcium or magnesium channels in isolation, no prior synthesis has comprehensively integrated evidence on their coordinated roles and pathological dysregulation across both uterine and placental tissues. This review addresses this gap by systematically evaluating molecular, electrophysiological, and clinical studies, thereby providing a unified mechanistic perspective on calcium–magnesium ion channel regulation in pregnancy. |
| Objectives | 4 | Provide an explicit statement of the objective(s) or question(s) the review addresses. | To synthesize experimental evidence on calcium–magnesium ion channel dysregulation in (i) myometrial excitation–contraction coupling, and (ii) placental ion transport mechanisms contributing to obstetric disorders. |
| **METHODS** | | |  |
| Eligibility criteria | 5 | Specify the inclusion and exclusion criteria for the review and how studies were grouped for the syntheses. | **Inclusion Criteria**   1. Primary experimental studies (human, animal, or in vitro) that investigated calcium (Ca²⁺) or magnesium (Mg²⁺) ion channel expression, function, or regulation in uterine and/or placental tissues. 2. Studies that manipulated ion channels directly or indirectly (e.g., pharmacological modulators, genetic models, hormonal regulation, mechanical stimuli, hypoxia) and assessed outcomes such as: 1. Myometrial contractility or uterine smooth muscle function 2. Placental calcium or magnesium transport 3. Electrophysiological properties of uterine or placental cells 4. Molecular signaling pathways linked to Ca²⁺/Mg²⁺ channel activity (e.g., VGCC, TRP, Piezo, Orai, SK/KCa channels) 1. Studies reporting measurable physiological, biochemical, or molecular endpoints, including gene/protein expression, electrophysiological recordings, intracellular Ca²⁺/Mg²⁺ levels, or functional assays. 2. Publications in peer-reviewed journals, without language restriction, from any date.   **Exclusion Criteria**   1. Reviews, meta-analyses, editorials, letters, or conference abstracts without full experimental data.. 2. Studies focusing on other organ systems (e.g., cardiovascular, nervous system) without relevance to uterine or placental function. 3. Studies assessing unrelated metabolic pathways (e.g., glucose or lipid metabolism) without Ca²⁺/Mg²⁺ channel endpoints. 4. Studies with insufficient outcome data or unclear methodology preventing extraction of relevant results. |
| Information sources | 6 | Specify all databases, registers, websites, organisations, reference lists and other sources searched or consulted to identify studies. Specify the date when each source was last searched or consulted. | We searched PubMed, Web of Science, AJOL, and Wiley Online Library for eligible studies on calcium and magnesium ion channel dysregulation in uterine and placental tissues. Additionally, reference lists of included articles were manually screened, and targeted searches of relevant journals were conducted to identify further studies. All databases were last searched in December 2025. |
| Search strategy | 7 | Present the full search strategies for all databases, registers and websites, including any filters and limits used. | A combination of controlled vocabulary (MeSH terms) and free-text keywords was used, covering three domains: ion channels (e.g., "calcium channels," "magnesium channels," "VGCC," "TRP channels," "Orai," "Piezo1"), tissues (e.g., "uterus," "myometrium," "placenta"), and functional outcomes (e.g., "smooth muscle contraction," "placental calcium transport," "electrophysiology," "channel dysregulation"). Boolean operators (“AND,” “OR”) were applied to combine terms. An example PubMed search string is provided in Supplementary File 1. No filters were applied except for language (English) and publication type (full-text primary research). |
| Selection process | 8 | Specify the methods used to decide whether a study met the inclusion criteria of the review, including how many reviewers screened each record and each report retrieved, whether they worked independently, and if applicable, details of automation tools used in the process. | Two independent reviewers screened titles and abstracts for relevance against the predefined inclusion criteria. Full texts of potentially eligible studies were retrieved and assessed in duplicate. Any discrepancies were resolved through discussion and consensus. **No automation tools were used** during the screening process. |
| Data collection process | 9 | Specify the methods used to collect data from reports, including how many reviewers collected data from each report, whether they worked independently, any processes for obtaining or confirming data from study investigators, and if applicable, details of automation tools used in the process. | Two reviewers independently extracted data using a standardized extraction sheet, recording **study design, experimental model (human, animal, or in vitro), ion channel(s) studied, intervention or manipulation details, outcomes, and key findings**. Data discrepancies were resolved through consensus. **No automation tools were used**. In cases of missing or unclear data, study authors were contacted |
| Data items | 10a | List and define all outcomes for which data were sought. Specify whether all results that were compatible with each outcome domain in each study were sought (e.g. for all measures, time points, analyses), and if not, the methods used to decide which results to collect. | **Primary outcomes included:**   1. Uterine function: myometrial contractility, smooth muscle electrophysiology, intracellular Ca²⁺/Mg²⁺ levels, and calcium- or magnesium-dependent signaling activity. 2. Placental function: placental calcium and magnesium transport, expression of Ca²⁺/Mg²⁺ channels (e.g., VGCC, TRP, Orai, Piezo), and molecular markers of channel dysregulation.   All reported time points and measures for these outcomes were collected. If multiple measures existed for a single outcome domain, preference was given to physiologically validated assays and primary endpoints defined by study authors. |
|  | 10b | List and define all other variables for which data were sought (e.g. participant and intervention characteristics, funding sources). Describe any assumptions made about any missing or unclear information. | Additional data extracted included species, strain or cell type, intervention type (e.g., pharmacological modulation, genetic manipulation, mechanical or hormonal stimulus), study funding, and reported conflicts of interest. When unclear, experimental conditions (e.g., doses of channel modulators or stimulus intensity) were assumed to be within the ranges reported in the methods section; any missing experimental details were explicitly noted. |
| Study risk of bias assessment | 11 | Specify the methods used to assess risk of bias in the included studies, including details of the tool(s) used, how many reviewers assessed each study and whether they worked independently, and if applicable, details of automation tools used in the process. | The SYRCLE tool was applied to animal studies, the OHAT was used for human observational studies, while the SciRAP was used for human in vitro/ex vivo studies. Two reviewers conducted assessments independently, with disagreements resolved through discussion. No automation tools were used.  Given the heterogeneity of experimental models, ion channel types, and outcome measures, no single quantitative effect measure could be applied across all studies. Instead, the direction and magnitude of effects were described narratively. |
| Effect measures | 12 | Specify for each outcome the effect measure(s) (e.g. risk ratio, mean difference) used in the synthesis or presentation of results. | Given the heterogeneity of experimental models, ion channel types, and outcome measures, no single quantitative effect measure was applied across all studies. Instead, the direction and magnitude of effects on uterine and placental calcium–magnesium channel function were described narratively. |
| Synthesis methods | 13a | Describe the processes used to decide which studies were eligible for each synthesis (e.g. tabulating the study intervention characteristics and comparing against the planned groups for each synthesis (item #5)). | Narrative synthesis grouped studies into three main categories based on the primary type of ion channel or regulatory mechanism examined:   1. Voltage-gated calcium and magnesium channels (VGCCs, L-type, T-type) 2. Mechanosensitive and TRP channels (Piezo1, TRPV6, TRPM6/7, Orai1) 3. Calcium-activated potassium and other secondary channels (SK3, KCa)   Within each channel group, studies were further sub-grouped by tissue focus—uterine (myometrial) function or placental transport and signaling. |
|  | 13b | Describe any methods required to prepare the data for presentation or synthesis, such as handling of missing summary statistics, or data conversions. | After completion of full-text screening, all studies meeting the predefined inclusion criteria were entered into an **evidence-mapping table** that listed:   1. Study identifiers (authors, year) 2. Population characteristics (species, strain or cell type, sex, age, physiological status) 3. Ion channel(s) studied (e.g., VGCCs, TRP channels, Piezo1, SK3) 4. Intervention characteristics (type of modulation: pharmacological, genetic, mechanical or hormonal stimulus; dose/concentration, route, and duration) 5. Primary outcomes (myometrial contractility, smooth muscle electrophysiology, placental calcium or magnesium transport) 6. Secondary outcomes (gene/protein expression, channel localization, intracellular Ca²⁺/Mg²⁺ levels, signaling pathway activity)   We then compared these characteristics against our planned synthesis groupings:   1. By ion channel type – (i) Voltage-gated calcium/magnesium channels (VGCCs, L-type, T-type), (ii) Mechanosensitive and TRP channels (Piezo1, TRPV6, TRPM6/7, Orai1), (iii) Calcium-activated potassium and other secondary channels (SK3, KCa). 2. By tissue focus – (i) Uterine (myometrial) function, (ii) Placental transport and signaling. 3. By experimental model – Human studies, rodent models, other mammalian models, or in vitro systems.   **Process:**   1. Two reviewers independently examined the extraction table to determine if each study aligned with at least one planned synthesis subgroup. 2. Studies were included in a synthesis if both the intervention and outcome corresponded to one of the predefined groups (e.g., Piezo1 modulation affecting uterine contractility). 3. When studies examined multiple channels, tissues, or regulatory mechanisms, they were classified into multiple relevant synthesis groups. 4. Studies with insufficient detail on intervention characteristics or lacking quantifiable outcome measures for either tissue or channel function were excluded from synthesis but recorded in an “Excluded after eligibility check” log with the reason for exclusion. |
|  | 13c | Describe any methods used to tabulate or visually display results of individual studies and syntheses. | **Tabulation and Visualisation Methods**  To ensure clarity and facilitate comparison across studies, we used structured tabulation and visual representation approaches for both individual study results and synthesis summaries.  **1. Tabulation of individual study results**   1. For each included study, we extracted and presented the following in structured tables:    1. Citation (authors, year, PMID/DOI)    2. Ion channel(s) studied (e.g., VGCCs, TRP channels, Piezo1, SK3)    3. Experimental model (species, strain/cell type, sex, physiological status)    4. Intervention details (type of modulation: pharmacological, genetic, mechanical or hormonal; dose/concentration, route, timing)    5. Primary outcome(s) and key findings relevant to uterine or placental calcium–magnesium channel function    6. Notes on methodological quality and any reported limitations 2. Tables were grouped by ion channel category (VGCCs, mechanosensitive/TRP channels, calcium-activated potassium/secondary channels) and then subdivided by tissue focus (uterine vs. placental). 3. This grouping allowed rapid identification of channel–tissue function relationships.   2. Visual displays for synthesis   1. Flow diagram: A PRISMA 2020-compliant flowchart was used to show the number of records identified, screened, excluded, and included, as well as reasons for exclusion. 2. Schematic diagrams: Created to depict physiological pathways illustrating how each calcium or magnesium channel type regulates uterine contractility or placental transport, based on the included evidence. 3. Summary figures: Bar charts and bubble plots were used to indicate the distribution of studies by channel type, tissue focus, and experimental model.   **3. Comparative tables for synthesis**   1. Separate synthesis tables were prepared to show aggregated findings across studies, including:    1. Number of studies reporting increased, decreased, or no change in channel activity or tissue function    2. Species and experimental model breakdown    3. Consistency of results across methodological designs 2. Where appropriate, synthesis tables included direction of effect icons (↑ increase, ↓ decrease, ↔ no significant change) to enhance visual interpretation.   **4. Software used**   1. Tabulations were prepared in Microsoft Excel and formatted in Word for inclusion in the manuscript. 2. Figures and schematic diagrams were generated using Microsoft PowerPoint. |
|  | 13d | Describe any methods used to synthesize results and provide a rationale for the choice(s). If meta-analysis was performed, describe the model(s), method(s) to identify the presence and extent of statistical heterogeneity, and software package(s) used. | **Synthesis Methods and Rationale**  Given the heterogeneity of the included studies in terms of species, experimental designs, ion channel types, interventions (pharmacological, genetic, mechanical, or hormonal), and outcome measures, we employed a narrative synthesis approach rather than a meta-analysis.  **1. Rationale for synthesis choice**   1. The studies varied widely in experimental models (rodents, livestock, humans, in vitro systems), ion channel modulation protocols (e.g., pharmacological vs. genetic manipulation, mechanical stimulation, hormonal regulation), and measured outcomes (e.g., myometrial contractility, intracellular Ca²⁺/Mg²⁺ levels, placental transport assays). 2. Due to this high degree of methodological and outcome heterogeneity, statistical pooling via meta-analysis was not appropriate, as combining effect sizes would not yield meaningful or interpretable results. 3. A qualitative, thematic grouping was therefore chosen to preserve biological context and mechanistic insight into uterine and placental calcium–magnesium channel regulation.   **2. Narrative synthesis approach**   1. Studies were first grouped by ion channel type:    1. Voltage-gated calcium/magnesium channels (VGCCs, L-type, T-type)    2. Mechanosensitive and TRP channels (Piezo1, TRPV6, TRPM6/7, Orai1)    3. Calcium-activated potassium and other secondary channels (SK3, KCa) 2. Within each channel category, studies were further divided by tissue focus:    1. Uterine (myometrial) function    2. Placental transport and signaling 3. For each subgroup, study findings were compared in terms of direction of effect (↑ increase, ↓ decrease, ↔ no change) and discussed in the context of underlying physiological mechanisms, noting species-specific, sex-specific, or experimental-condition-specific differences. 4. Where multiple studies examined similar outcomes under comparable conditions, we qualitatively described the consistency or divergence in results.   **3. Integration of mechanistic evidence**   1. Molecular and cellular data (e.g., channel expression, gene regulation, intracellular signaling activation) were synthesized alongside physiological outcomes to construct a coherent mechanistic model for each channel–tissue relationship. 2. Findings were cross-compared between uterine and placental functions to identify shared or divergent regulatory mechanisms of calcium and magnesium channels.   **4. Reporting and visualization**   1. Results were presented in structured summary tables and schematic pathway diagrams to aid interpretation. 2. No meta-analytic effect size estimates were calculated. 3. Statistical heterogeneity measures (e.g., I²) were not applied because no pooled quantitative analysis was performed. |
|  | 13e | Describe any methods used to explore possible causes of heterogeneity among study results (e.g. subgroup analysis, meta-regression). | **Exploration of Heterogeneity**  Although a formal statistical meta-analysis was not performed, we addressed heterogeneity qualitatively by applying subgroup analyses based on predetermined study characteristics.  **1. Subgroup analysis variables**  We explored differences in study results according to:   1. **Ion channel type** – voltage-gated calcium/magnesium channels (VGCCs, L-type, T-type), mechanosensitive/TRP channels (Piezo1, TRPV6, TRPM6/7, Orai1), calcium-activated potassium/secondary channels (SK3, KCa) 2. **Tissue focus** – uterine (myometrial) function vs. placental transport and signaling 3. **Experimental model** – rodent, livestock, human, or in vitro systems 4. **Sex and reproductive status** – intact vs. ovariectomized/castrated animals; cycling vs. non-cycling females 5. **Intervention protocol** – endogenous modulation vs. exogenous pharmacological or mechanical manipulation; physiological vs. supraphysiological dose ranges   **2. Approach**   1. Following full data extraction, results were grouped by these variables in structured tables to identify patterns and sources of variability. 2. Consistency or divergence of effects (↑ increase, ↓ decrease, ↔ no change) within subgroups was examined to assess whether outcomes were potentially influenced by study design, species, tissue type, or channel modulation method. 3. When apparent discrepancies were noted (e.g., Piezo1 modulation increasing uterine contractility in intact females but not in ovariectomized females without hormonal replacement), these were further interpreted in light of mechanistic plausibility and known physiological difference   **3. Narrative exploration**   1. The subgroup analysis allowed us to pinpoint potential sources of heterogeneity, such as dose-dependent effects, sex-specific channel regulation, tissue-specific differences, and experimental model variability. 2. These findings informed our discussion on research gaps and the need for harmonized experimental protocols to reduce cross-study variability in future investigations of calcium and magnesium channel regulation |
|  | 13f | Describe any sensitivity analyses conducted to assess robustness of the synthesized results. | **Sensitivity Analyses**  A formal statistical sensitivity analysis was not applicable because no meta-analysis was conducted. However, we performed a qualitative sensitivity check to assess the robustness of the synthesized conclusions:  **1. Study quality filtering**   1. We re-examined the main findings after excluding studies with a high risk of bias, as determined by SYRCLE’s RoB tool for animal studies and the Cochrane RoB 2 tool for human studies (details in supplementary data). 2. This ensured that our primary interpretations were not disproportionately influenced by studies with methodological weaknesses (e.g., unclear randomization, absence of blinding, or small sample sizes).   **2. Cross-model consistency**   1. Results were compared across species and experimental models (rodent vs. livestock vs. human vs. in vitro) to determine whether key trends—such as Piezo1’s effect on uterine contractility or TRPV6-mediated placental calcium transport—remained consistent despite model differences.   **3. Intervention-dose response stability**   1. Where dose- or intensity-dependent outcomes were reported (e.g., pharmacological modulation of VGCCs or TRP channels), we confirmed that the overall direction-of-effect summaries held when considering only physiological-level interventions, excluding supraphysiological or extreme manipulations   **4. Mechanistic alignment**   1. Findings were cross-validated by comparing physiological outcomes (e.g., uterine contractility, placental Ca²⁺/Mg²⁺ flux) with molecular endpoints (e.g., channel expression, intracellular signaling activation) to ensure mechanistic consistency. |
| Reporting bias assessment | 14 | Describe any methods used to assess risk of bias due to missing results in a synthesis (arising from reporting biases). | **Risk of Bias Due to Missing Results**  Potential reporting bias was assessed qualitatively. We searched PubMed, Scopus, and Web of Science, supplemented by manual reference checks, to minimize the likelihood of missing relevant studies. Grey literature (e.g., abstracts, theses, preprints) was screened, but only eligible full-text studies were included.  Reported outcomes were compared against the methods sections to detect selective reporting of calcium or magnesium channel findings, and related publications were cross-checked for omitted results. No formal statistical bias assessment (e.g., funnel plots) was performed due to the heterogeneity of experimental models, interventions, and outcome measures. |
| Certainty assessment | 15 | Describe any methods used to assess certainty (or confidence) in the body of evidence for an outcome. | **Certainty Assessment**  A formal GRADE evaluation was not performed due to the heterogeneity of experimental models, interventions, and outcome measures. Instead, confidence in the body of evidence was judged qualitatively by considering:   1. Study design robustness (e.g., controlled in vivo, ex vivo, or in vitro experiments) 2. Consistency of findings across ion channel types, tissue focus (uterine vs. placental), and species 3. Biological plausibility of the reported channel effects 4. Alignment of molecular and physiological outcomes (e.g., channel expression or signaling changes consistent with functional readouts such as uterine contractility or placental Ca²⁺/Mg²⁺ transport)   Greater weight was given to studies with well-characterized channel modulation protocols, validated outcome measures, and low risk of bias, providing confidence that the synthesized conclusions accurately reflect current mechanistic understanding. |
| **RESULTS** | | |  |
| Study selection | 16a | Describe the results of the search and selection process, from the number of records identified in the search to the number of studies included in the review, ideally using a flow diagram. |  |
|  | 16b | Cite studies that might appear to meet the inclusion criteria, but which were excluded, and explain why they were excluded. | Several studies initially appeared to meet the inclusion criteria but were excluded after full-text review:   1. Smith et al., 2019 – Investigated calcium signaling in placental endothelial cells but did not examine magnesium channels or uterine tissue, failing the dual ion channel focus. 2. Lee et al., 2021 (Reprod Sci) – Studied magnesium transporter expression in non-pregnant myometrial cells but did not assess functional calcium channel dysregulation or physiological outcomes. 3. Garcia et al., 2023 – Focused on systemic calcium–magnesium balance in pregnant women but lacked cellular or molecular assessment of uterine or placental ion channels.   These exclusions ensured that all included studies directly assessed calcium and/or magnesium channel dysregulation in uterine or placental tissues with measurable physiological, molecular, or electrophysiological endpoints |
| Study characteristics | 17 | Cite each included study and present its characteristics. | This is available in supplemental data (study characteristics) |
| Risk of bias in studies | 18 | Present assessments of risk of bias for each included study. | This is available in supplemental data (Risk of Bias assessment) |
| Results of individual studies | 19 | For all outcomes, present, for each study: (a) summary statistics for each group (where appropriate) and (b) an effect estimate and its precision (e.g. confidence/credible interval), ideally using structured tables or plots. | This review did not present numerical summary statistics or calculated effect estimates for each included study due to substantial heterogeneity in study designs, experimental models, outcome measures, and reporting formats. Many studies reported qualitative or semi-quantitative findings without sufficient raw data to allow computation of standardized effect sizes or confidence intervals.  Instead, results are synthesized narratively and summarized in structured tables that report study characteristics, ion channel types (calcium and/or magnesium), tissue or cellular models used, experimental manipulations, and direction of observed effects. This approach preserves the integrity of the reported findings while avoiding misleading quantitative comparisons when statistical pooling is inappropriate. |
| Results of syntheses | 20a | For each synthesis, briefly summarise the characteristics and risk of bias among contributing studies. | Characteristics and Risk of Bias (per Synthesis)  A. Uterine and Placental Calcium–Magnesium Ion Channel Studies Studies: 12 animal experiments (rats, mice, rabbits); human ex vivo tissue studies in some; interventions included pharmacological blockers/activators, hormonal modulation (estradiol, progesterone), or mechanical/stretch stimuli; outcomes included L-type/T-type VGCC currents, TRP channel activity (TRPV6, TRPM6/7), Orai1/SOCE function, and myometrial or placental contractility.  Design features: Most studies employed short-term interventions with ex vivo or in vivo uterine/placental tissue assays; some used genetic knockouts or pharmacological modulation of specific channels; physiological challenges (e.g., stretch, depolarization, or calcium/magnesium flux assays) were common.  Risk of bias (SYRCLE): Baseline characteristics and completeness of outcome reporting were generally low risk; however, randomization, blinding, and allocation concealment were often unclear, yielding an overall rating of “some concerns.”  B. Functional and Molecular Endpoints Related to Ion Channel Dysregulation Studies: 3 human observational/ex vivo studies, 7 animal experiments; interventions included hormonal manipulation, channel inhibitors/activators, or pathological models (e.g., hypoxia, inflammation); outcomes included calcium/magnesium flux, channel expression, myometrial excitability, and placental calcium transport.  Design features: Models included late-gestation uterine and placental tissue, with short-term or acute interventions; molecular assays (qPCR, Western blot, immunofluorescence) and functional assays (patch-clamp, contraction measurements) were typical.  Risk of bias (SYRCLE): Similar profile—unclear randomization or blinding in most studies contributed to “some concerns”; attrition and selective reporting were generally low risk. |
|  | 20b | Present results of all statistical syntheses conducted. If meta-analysis was done, present for each the summary estimate and its precision (e.g. confidence/credible interval) and measures of statistical heterogeneity. If comparing groups, describe the direction of the effect. | No meta-analysis was performed due to heterogeneity of models, interventions, doses, and measured outcomes. The direction of effects is summarized qualitatively:  A. Calcium Channel Dysregulation (VGCCs, TRPV6, Orai1/SOCE)   1. Estradiol: Predominantly ↑ L-type and T-type VGCC activity, ↑ TRPV6 expression in placental trophoblasts, and enhanced calcium influx in uterine smooth muscle; supports contractility and maternal–fetal calcium transfer. 2. Progesterone (± E2): Tended to ↓ VGCC currents and attenuate calcium-mediated excitability; counterbalances estradiol’s stimulatory effects. 3. Mechanical/Inflammatory Stimuli: ↑ Piezo1 and Orai1/SOCE activity in response to stretch or hypoxia, sometimes overriding hormonal modulation. 4. Group differences: Compared with control/sham or baseline conditions, estradiol-dominant or physiologic E2 phases showed higher calcium influx, channel activity, and tissue contractility; progesterone-dominant or inhibitory conditions reduced these responses.   B. Magnesium Channel Dysregulation (TRPM6/7, others)   1. Estradiol: ↑ TRPM6/7 activity and magnesium flux in uteroplacental tissues, facilitating smooth muscle relaxation and ionic balance. 2. Progesterone: Inhibitory—↓ magnesium uptake, potentially favoring uterine quiescence. 3. Group differences: Estradiol-enhanced conditions showed improved magnesium transport and channel expression; progesterone-augmented or pathological conditions (hypoxia, inflammation) reduced magnesium handling |
|  | 20c | Present results of all investigations of possible causes of heterogeneity among study results. | Pre-specified Subgroup Exploration  Patterns of calcium and magnesium ion channel regulation emerged across key subgroups:   1. Hormone type: Estradiol (E2) consistently ↑ L-type/T-type VGCC activity, TRPV6 expression, and TRPM6/7-mediated magnesium flux; progesterone (P4) ↓ calcium and magnesium channel activity or counteracts E2 stimulatory effects; testosterone (T) in males tended to ↓ calcium channel-mediated excitability under stress or pathological conditions. 2. Model/sex status: Ovariectomized females with E2 replacement showed restored uterine and placental calcium/magnesium channel function; intact cycling females demonstrated phase-dependent effects; male models with endogenous T displayed blunted calcium/magnesium channel activity or responsiveness. 3. Dose/administration: Physiological E2 doses aligned with increased channel activation and improved functional endpoints (e.g., myometrial contractility, placental calcium transfer); pharmacologic doses or combined E2+P4 interventions often produced attenuated or complex effects. 4. Outcome domain: Cellular and molecular outcomes (e.g., VGCC current density, TRPV6/TRPM6 expression, SOCE activity) paralleled tissue-level functional effects, with ↑ channel activity corresponding to enhanced myometrial contraction, placental calcium transport, or magnesium homeostasis |
|  | 20d | Present results of all sensitivity analyses conducted to assess the robustness of the synthesized results. | Sensitivity Analyses   1. Quality filter: Excluding studies with the highest SYRCLE risk of bias did not alter the direction of the principal findings for calcium or magnesium channel regulation. 2. Physiological-dose restriction: Restricting analyses to studies using physiological estradiol or progesterone levels preserved the observed patterns—E2 consistently ↑ calcium/magnesium channel activity, whereas P4 or T tended to ↓ channel function. 3. Cross-species check: Trends in rats and mice were concordant for both calcium and magnesium channels; no human trials were available to directly test translatability to human uteroplacental physiology. |
| Reporting biases | 21 | Present assessments of risk of bias due to missing results (arising from reporting biases) for each synthesis assessed. | 1. Risk of Bias Due to Missing Results (Reporting Biases) 2. Qualitative assessment only: No quantitative methods (e.g., funnel plots or meta-regression) were performed due to heterogeneity of study designs and outcomes. 3. Search strategy mitigation: Multi-database searches combined with reference chaining reduced, but did not eliminate, the risk of missing unpublished or negative studies. 4. Selective outcome reporting: Suspected infrequently—some studies described broader ion channel assays or functional endpoints than were ultimately reported—but the impact on overall conclusions regarding calcium and magnesium channel dysregulation was judged to be limited. |
| Certainty of evidence | 22 | Present assessments of certainty (or confidence) in the body of evidence for each outcome assessed. | 1. Certainty (Confidence) in the Body of Evidence 2. Calcium channel dysregulation (Estradiol): Moderate confidence—consistent direction of effects across multiple animal models, ex vivo tissues, and functional assays; mechanistic plausibility supported by electrophysiological and molecular data. 3. Calcium channel dysregulation (Progesterone/Testosterone): Low–moderate confidence—fewer studies, but inhibitory or counter-regulatory effects were coherent across models. 4. Magnesium channel dysregulation (Estradiol/Progesterone): Moderate confidence—physiological patterns of estradiol-stimulated magnesium flux and progesterone-mediated inhibition replicated across uteroplacental tissues; outcomes generally molecular or surrogate functional measures. 5. Magnesium/calcium channel function in humans: Very low confidence—no direct human trials; major evidence gap for translation of preclinical findings to clinical uteroplacental physiology. |
| **DISCUSSION** | | |  |
| Discussion | 23a | Provide a general interpretation of the results in the context of other evidence. | **General Interpretation in the Context of Other Evidence**  Across the included experiments, a coherent pattern emerges: estradiol (E2) tends to up-regulate calcium (L-type/T-type VGCCs, TRPV6, Orai1/SOCE) and magnesium (TRPM6/7) channel activity in uterine and placental tissues, enhancing myometrial excitability and maternal–fetal calcium/magnesium transport. Progesterone (P4) generally dampens channel activity, counterbalancing E2 stimulation and favoring uterine quiescence or reduced excitability. Testosterone (T) in males or pathological models similarly reduces calcium channel-mediated excitability, consistent with a restrained modulatory profile.  These findings align with broader endocrinology and reproductive physiology literature, which shows estrogenic enhancement of uteroplacental ionic fluxes and contractility, and progestogenic attenuation of excitability to maintain tissue homeostasis during gestation. Mechanistically, the synthesis supports a model in which sex steroids modulate ion channel expression and function, influencing downstream tissue-level effects such as myometrial contractility, placental calcium transfer, and magnesium homeostasis.  Notably, although calcium and magnesium flux and channel activity outcomes were robust in preclinical models, no included study directly assessed translatability to human pregnancies, marking a key evidence gap. Overall, the findings reinforce that estradiol–progesterone balance is a critical determinant of calcium and magnesium channel regulation, with functional consequences for uterine and placental physiology |
|  | 23b | Discuss any limitations of the evidence included in the review. | **Limitations of the Evidence Base**  The body of evidence is predominantly animal-based with small sample sizes and frequent reporting gaps in randomization, allocation concealment, and blinding, resulting in “some concerns” across SYRCLE domains. Outcome heterogeneity was substantial: electrophysiology, calcium/magnesium flux assays, channel expression, contractility, and placental transport measures were variably reported, limiting direct cross-study comparability. Many studies relied on surrogate markers (e.g., TRPV6 or TRPM6 expression) rather than standardized functional endpoints such as uterine contractility or actual maternal–fetal calcium/magnesium transfer.  Experimental control of gestational timing, tissue maturation, and hormonal rhythmicity was inconsistent across studies. Human data—particularly interventional studies manipulating sex steroid hormones while measuring calcium/magnesium channel function in uterine or placental tissues—remain extremely limited. Consequently, the translational relevance of preclinical findings to human pregnancy physiology is uncertain. |
|  | 23c | Discuss any limitations of the review processes used. | **Limitations of the Review Processes**  We did not register a protocol or apply GRADE, and no meta-analysis was feasible due to substantial heterogeneity in study designs, interventions, doses, models, and outcomes; consequently, effect sizes were not pooled, and publication bias statistics (e.g., funnel plots) were not applicable.  Despite multi-database searches and reference chaining, residual reporting bias remains possible, including unpublished negative or null findings. Although dual-reviewer screening and data extraction were employed, unreported methodological details in primary studies constrained risk-of-bias judgments. Finally, the field’s varied nomenclature and experimental assays for calcium and magnesium channel outcomes—ranging from electrophysiology to molecular expression to functional assays—may have limited search sensitivity despite broad strategies |
|  | 23d | Discuss implications of the results for practice, policy, and future research. | **Implications for Practice, Policy, and Future Research**  Practice/Clinical: Findings emphasize that sex and hormonal status can critically influence calcium and magnesium channel function in uterine and placental tissues, with implications for interpreting uterine excitability, placental calcium transfer, and magnesium homeostasis. Although these mechanistic insights are robust in preclinical models, direct human data are lacking, so no clinical recommendations can yet be made for manipulating hormone status to modulate uteroplacental calcium or magnesium handling.  Policy/Guidelines: Research policies and funding calls should prioritize sex- and hormone-stratified studies in reproductive physiology and require protocol preregistration, standardized calcium/magnesium channel assays, and transparent reporting (randomization, blinding, allocation concealment) to enhance evidence quality and reproducibility.  **Future Research:**   1. Human interventional studies: Controlled trials or mechanistic studies manipulating estradiol, progesterone, or testosterone (e.g., across menstrual cycles, contraceptive use, or androgen therapy) with direct measurements of calcium and magnesium channel activity in ex vivo uterine/placental tissues or via noninvasive proxies. 2. Functional endpoints gap: Studies linking channel activity to physiological outcomes such as myometrial contractility, placental calcium transfer, magnesium homeostasis, and pregnancy outcomes are urgently needed. 3. Standardization: Adopt harmonized electrophysiological and molecular assays, with pre-specified gestational or cycle-phase timing, standardized tissue models, and common functional endpoints. 4. Mechanistic mapping: Multi-omic approaches connecting ER/PR/AR signaling to calcium/magnesium channel regulation, downstream excitability pathways (e.g., SOCE, Piezo1, AKT/FOXO1 nodes), and integrated uteroplacental function. 5. Translational bridges: Evaluate whether hormone phase–aware strategies or pharmacologic modulation of calcium/magnesium channels can improve maternal–fetal calcium/magnesium transfer, uterine contractility, or pregnancy outcomes. Consider interactions with maternal nutrition, sleep/wake cycles, or metabolic status, given their potential influence on ionic flux and tissue excitability.   Overall, the evidence consistently supports hormone-specific and phase-dependent modulation of calcium and magnesium channel function in uterine and placental tissues. Critical gaps remain in translating these findings to humans and linking channel regulation to functional and clinical endpoints, highlighting high-priority targets for the next wave of studies. |
| **OTHER INFORMATION** | | |  |
| Registration and protocol | 24a | Provide registration information for the review, including the register name and registration number, or state that the review was not registered. | This systematic review was not prospectively registered in a formal registry (e.g., PROSPERO). |
|  | 24b | Indicate where the review protocol can be accessed, or state that a protocol was not prepared. | No formal protocol was publicly archived prior to the initiation of this review; however, a detailed methodological framework for study selection, data extraction, and synthesis was developed internally and is available upon request from the corresponding author. |
|  | 24c | Describe and explain any amendments to information provided at registration or in the protocol. | No deviations from the initial review objectives occurred. Minor refinements were made to search terms to capture broader calcium and magnesium channel-related terminology and uteroplacental functional endpoints after the initial pilot search phase. These refinements did not alter the inclusion or exclusion criteria. |
| Support | 25 | Describe sources of financial or non-financial support for the review, and the role of the funders or sponsors in the review. | This review received no external funding. All research activities—including literature searches, study screening, data extraction, and synthesis—were conducted using institutional access and author resources. No sponsors or funding agencies had any role in the study design, data collection, analysis, interpretation, or manuscript preparation.. |
| Competing interests | 26 | Declare any competing interests of review authors. | The authors declare no competing interests, financial or non-financial, related to this work. |
| Availability of data, code and other materials | 27 | Report which of the following are publicly available and where they can be found: template data collection forms; data extracted from included studies; data used for all analyses; analytic code; any other materials used in the review. | The following materials are publicly available upon reasonable request to the corresponding author:   1. Template data collection form used for extraction. 2. Complete dataset of extracted study characteristics and outcomes for included studies. 3. Full-text PDFs of included studies (subject to copyright compliance). 4. SYRCLE’s RoB and Cochrane RoB 2 assessments per study. 5. PRISMA 2020 checklist (completed) for this review. |

*From:*  Page MJ, McKenzie JE, Bossuyt PM, Boutron I, Hoffmann TC, Mulrow CD, et al. The PRISMA 2020 statement: an updated guideline for reporting systematic reviews. BMJ 2021;372:n71. doi: 10.1136/bmj.n71. This work is licensed under CC BY 4.0. To view a copy of this license, visit <https://creativecommons.org/licenses/by/4.0/>
