# Supplementary material for: Utero-placental calcium and magnesium ion channels: A systematic review of obstetric implications of their alterations
Source: Channels (Austin). 2026 Aug 26;20(1):2724674. doi: 10.1080/19336950.2026.2724674 (PMC13523982; doi:10.1080/19336950.2026.2724674)
Supplement: Supplementary file 2 [file KCHL_A_2724674_SM2553.docx]

**Supplementary File S2: Search strings used for each database**

| **Database** | **Platform / Interface** | **Search String** | **Date searched** |
| --- | --- | --- | --- |
| **PubMed/MEDLINE** | NCBI | (("calcium" OR "Ca²⁺" OR "magnesium" OR "Mg²⁺" OR "calcium channels" OR "L-type calcium channel" OR "T-type calcium channel" OR "TRP channels" OR "TRPC" OR "TRPV" OR "TRPM" OR "TRPV4" OR "TRPV6" OR "TRPM6" OR "TRPM7" OR "MagT1" OR "SLC41A1" OR "store-operated calcium entry" OR "SOCE" OR "CRAC" OR "Orai1" OR "STIM1" OR "Piezo1" OR "BKCa" OR "SK3" OR "KATP" OR "IP3 receptor" OR "ryanodine receptor" OR "calcium-sensing receptor" OR "CaSR") AND ("uterus" OR "myometrium" OR "placenta" OR "trophoblast" OR "syncytiotrophoblast") AND ("pregnancy" OR "labor" OR "parturition" OR "preterm labor" OR "preeclampsia" OR "uterine atony" OR "fetal growth restriction")) | 2^nd^ April, 2026 |
| **Web of Science** | Clarivate | TS=(("calcium" OR "Ca²⁺" OR "magnesium" OR "Mg²⁺" OR "calcium channels" OR "L-type calcium channel" OR "T-type calcium channel" OR "TRP channels" OR "TRPC" OR "TRPV" OR "TRPM" OR "TRPV4" OR "TRPV6" OR "TRPM6" OR "TRPM7" OR "MagT1" OR "SLC41A1" OR "store-operated calcium entry" OR "SOCE" OR "CRAC" OR "Orai1" OR "STIM1" OR "Piezo1" OR "BKCa" OR "SK3" OR "KATP" OR "IP3 receptor" OR "ryanodine receptor" OR "calcium-sensing receptor" OR "CaSR") AND ("uterus" OR "myometrium" OR "placenta" OR "trophoblast" OR "syncytiotrophoblast") AND ("pregnancy" OR "labor" OR "parturition" OR "preterm labor" OR "preeclampsia" OR "uterine atony" OR "fetal growth restriction")) | 2^nd^ April, 2026 |
| **AJOL** | African Journals Online | (("calcium" OR "Ca²⁺" OR "magnesium" OR "Mg²⁺" OR "calcium channels" OR "L-type calcium channel" OR "T-type calcium channel" OR "TRP channels" OR "TRPC" OR "TRPV" OR "TRPM" OR "TRPV4" OR "TRPV6" OR "TRPM6" OR "TRPM7" OR "MagT1" OR "SLC41A1" OR "store-operated calcium entry" OR "SOCE" OR "CRAC" OR "Orai1" OR "STIM1" OR "Piezo1" OR "BKCa" OR "SK3" OR "KATP" OR "IP3 receptor" OR "ryanodine receptor" OR "calcium-sensing receptor" OR "CaSR") AND ("uterus" OR "myometrium" OR "placenta" OR "trophoblast" OR "syncytiotrophoblast") AND ("pregnancy" OR "labor" OR "parturition" OR "preterm labor" OR "preeclampsia" OR "uterine atony" OR "fetal growth restriction")) | 2^nd^ April, 2026 |
| **Wiley Online Library** | Wiley | (("calcium" OR "Ca²⁺" OR "magnesium" OR "Mg²⁺" OR "calcium channels" OR "L-type calcium channel" OR "T-type calcium channel" OR "TRP channels" OR "TRPC" OR "TRPV" OR "TRPM" OR "TRPV4" OR "TRPV6" OR "TRPM6" OR "TRPM7" OR "MagT1" OR "SLC41A1" OR "store-operated calcium entry" OR "SOCE" OR "CRAC" OR "Orai1" OR "STIM1" OR "Piezo1" OR "BKCa" OR "SK3" OR "KATP" OR "IP3 receptor" OR "ryanodine receptor" OR "calcium-sensing receptor" OR "CaSR") AND ("uterus" OR "myometrium" OR "placenta" OR "trophoblast" OR "syncytiotrophoblast") AND ("pregnancy" OR "labor" OR "parturition" OR "preterm labor" OR "preeclampsia" OR "uterine atony" OR "fetal growth restriction")) | 2^nd^ April, 2026 |
